# New Insights into Intestinal Permeability in Irritable Bowel Syndrome-Like Disorders: Histological and Ultrastructural Findings of Duodenal Biopsies

**DOI:** 10.3390/cells10102593

**Published:** 2021-09-29

**Authors:** Selenia Miglietta, Raffaele Borghini, Michela Relucenti, Veronica Sorrentino, Rui Chen, Xiaobo Li, Francesco Fazi, Giuseppe Donato, Giuseppe Familiari, Vincenzo Petrozza, Antonio Picarelli

**Affiliations:** 1 Department of Anatomical, Histological, Forensic and Orthopedic Sciences, Sapienza University of Rome, 00161 Rome, Italy; michela.relucenti@uniroma1.it (M.R.); francesco.fazi@uniroma1.it (F.F.); giuseppe.familiari@uniroma1.it (G.F.); 2 Department of Translation and Precision Medicine, Sapienza University of Rome, 00161 Rome, Italy; giuseppe.donato@uniroma1.it (G.D.); antonio.picarelli@uniroma1.it (A.P.); 3 Department of Medico-Surgical Sciences and Biotechnologies, Sapienza University of Rome, 00161 Rome, Italy; sorre.vs@gmail.com (V.S.); vincenzo.petrozza@uniroma1.it (V.P.); 4 Key Laboratory of Environmental Medicine Engineering, Ministry of Education, School of Public Health, Southeast University, Nanjing 218889, China; ruichen@ccmu.edu.cn (R.C.); 101011116@seu.edu.cn (X.L.)

**Keywords:** celiac disease, wheat allergy, non-celiac gluten sensitivity, nickel allergy, IBS-like disorders, diagnosis, transmission electron microscopy (TEM), intestinal permeability, ultrastructure, duodenal biopsies

## Abstract

Background and Aim: Diarrhea, abdominal pain, and bloating are frequent in irritable bowel syndrome (IBS)-like disorders, although little is known about their intestinal ultrastructural alterations. The aim of the present study was to study duodenal biopsies from IBS-like patients to find ultrastructural alterations. Materials and Methods: Study design: descriptive comparative pilot study. Thirty outpatients (9 male and 21 female; median age 37.7 years; range, 20 to 65 years) complaining of IBS-like symptoms were enrolled between January 2015 to May 2019 and were divided into 6 groups, each equally consisting of 5 patients: (A) untreated celiac disease (uCD); (B) treated celiac disease (tCD); (C) wheat allergy (WA); (D) Non-celiac gluten sensitivity (NCGS); (E) Nickel allergic contact mucositis (Ni ACM); (F) controls affected by GERD. Transmission electron microscopy (TEM) morphological characteristics were: microvilli length, intermicrovillar distance, junctional complexes (JC) gap width, autophagic bodies, apoptosis, altered mitochondria, lipid/chylomicron droplets, and mast cells. Regarding JC, we focused on tight junctions (TJ), adherens junctions (AJ), and desmosomes. Results: Major alterations in microvilli length and intermicrovillar distance have been observed in the subjects affected by uCD. Microvilli of tCD patients showed marked recovery after adequate GFD, although not comparable to controls. Intermediate microvillar alterations were instead observed in NCGS and Ni ACM, while characteristics of WA subjects appeared more similar to tCD. Regarding JC, TJ did not show significant differences between all groups studied, including controls. The AJ were significantly more dilated in all groups compared to controls, while no significant differences were found between the pathological groups. The distance between desmosomes was greater in uCD, NCGS, and Ni ACM than in tCD, WA, and controls. Finally, intracellular alterations have been detected in most of the groups studied although they seemed more unspecific. Conclusions: TEM analysis confirmed damages to the intestinal barrier and defense mechanisms by enterocytes in IBS-like patients, probably linked to low-grade inflammation or adverse reactions triggered by food allergens, heavy metals, or other unknown. On the other hand, our study needs confirmation and further investigations with larger populations to facilitate diagnosis, therapy, and prevention of IBS-like disorders in the future.

## 1. Introduction

More and more patients refer to gastroenterologists complaining of symptoms, such as chronic diarrhea, constipation, abdominal pain, and bloating, which may be observed in Irritable Bowel Syndrome (IBS) [1]. Adverse reactions to foods and other gastrointestinal disorders often present clinical features which may overlap with IBS and can be thus called “IBS-like disorders” [2]. Although IBS has an estimated high prevalence, its etiopathogenetic mechanisms have been still poorly understood [1,3]. Celiac Disease (CD), Non-celiac Gluten Sensitivity (NCGS), Wheat Allergy (WA), and Nickel Allergic Contact Mucositis (Ni ACM) are the most common IBS-like disorders, although their specific intestinal alterations and their etiopathogenetic mechanisms are not well-known [2]. 

Recently, the scientific world has been focusing on intestinal ultrastructural alterations to find the causes of IBS-like clinical pictures [4]. In the last decades, CD has become a paradigm of chronic intestinal inflammation where the loss of duodenal mucosa barrier function is affected by genetics, proteomics, and other possible factors [5]. Consequently, we can hypothesize that similar ultrastructural barrier alterations may also be present in other IBS-like disorders.

The apical junctional complexes (AJC) are important components of the intestinal barrier and are located at the apex of the lateral membrane of epithelial cells. The AJC includes tight junctions (TJ), adherens junctions (AJ), and desmosomes [6,7]. When the integrity of this epithelial barrier is disrupted, specific pro-inflammatory pathways are activated [8,9].

To date, there is no gold standard diagnostic tool to measure the intestinal barrier function, therefore an attempt has been made to use and combine different methods in vivo, in vitro, and ex vivo [10].

Not only functional, but also structural and molecular alterations have been described, especially for CD, and although similar suggestions have also been proposed for NCGS and other clinical conditions, there is still a lack of knowledge on disease-specific duodenal ultrastructural alterations and the consequent increased intestinal permeability [11]. Below we analyze the most common IBS-like disorders.

### 1.1. Celiac Disease

CD is an autoimmune reaction to ingested gluten in genetically predisposed subjects, has an estimated prevalence of 1% and the main target organ is the small bowel [12]. CD diagnosis is based on specific serologic markers and villous atrophy on histologic examination of duodenal biopsies during a gluten-containing diet [13]. The resolution of symptoms and the recovery of histological abnormalities can be obtained only after lifelong strict adherence to a gluten-free diet (GFD) [14,15].

Ultrastructural alterations were already observed in duodenal biopsies of untreated CD patients in the 1960s: in these subjects, the microvilli of the epithelial surface were reduced in height and showed considerable irregularities in both shape and intermicrovillar distance [16]. Subsequent studies by TEM have then highlighted a saccular dilation/destruction of the TJ, which returned to normal after an appropriate GFD. However, these data on ultrastructural changes did not reveal a disease specificity, as they were also observed in other conditions and are not related to the intensity of mucosal inflammation [4].

### 1.2. Wheat Allergy (WA)

WA is an IgE-mediated reaction with a prevalence rate of 0.2–0.9%. Signs and symptoms of WA may affect the respiratory tract, the skin, or the gastrointestinal tract. WA diagnosis is based on Skin Prick Tests, specific in vitro IgE, and functional tests. In recent years, a flow cytometry-assisted Basophil Activation Test (BAT) has been introduced as an in vitro functional test for the diagnosis of immediate allergy: it is a good alternative for those patients at risk of severe anaphylactic reactions or with doubtful test results [17]. Unlike CD, WA does not seem to cause permanent gastrointestinal damage. A strict wheat-free diet is recommended [2].

### 1.3. Non-Celiac Gluten Sensitivity (NCGS)

NCGS is characterized by gastrointestinal and extra-intestinal symptoms triggered by gluten consumption. To date, there are no specific markers for NCGS [18,19].

Although there is no gold standard, NCGS diagnosis can be suggested after a double-blind placebo-controlled gluten challenge or after a specific gluten oral mucosa patch test [18,20]. Other immunohistochemical markers proposed still appear rather unspecific and need further investigation [18,21].

### 1.4. Nickel Allergic Contact Mucositis (Ni ACM)

A Ni-rich diet in Ni-sensitive patients has recently been identified as one of the most frequent causes of IBS-like symptoms [2]. Ni has a ubiquitous nature and its possible routes of exposure are inhalation, skin absorption and ingestion. It is contained in many foods, especially fruits and vegetables [22], and their ingestion seems to cause the so-called “Systemic nickel allergy syndrome (SNAS)” in predisposed subjects. SNAS can clinically manifest as Allergic Contact Mucositis (ACM), whose prevalence is estimated to even exceed 30% [23]. A specific Ni oral mucosal patch test (Ni omPT) has been proposed for the diagnosis of Ni ACM. Ni omPT can be further supported by Laser Doppler Perfusion Imaging (LDPI) [24]. Duodenal biopsies from Ni-sensitive patients usually show normal histological results or mild unspecific alterations (type 0 or I according to Marsh–Oberhuber) [25].

We aimed to identify specific duodenal mucosa ultrastructural features and alterations in IBS-like disorders utilizing Transmission Electron Microscopy (TEM), to shed some light on these common pathologic conditions.

## 2. Materials and Methods

### 2.1. Study Participants

Study design: observational comparative pilot study. Thirty outpatients (9 males and 21 females; median age 37.7 years; range, 20 to 65 years) complaining of IBS-like symptoms and referring to Gastroenterology Unit—Department of Translation and Precision Medicine, were enrolled between January 2015 to May 2019. In order to avoid potential sources of bias, patients have been consecutively included in specific groups according to diagnosis until we reached the predetermined and equal number of 5 patients for each group. Exclusion criteria were: history of past or current cancer; inflammatory bowel disease; infectious diseases.

All procedures followed in this study were made for diagnostic purposes. The study has been performed following the ethical principles of the 1975 Helsinki Declaration of the World Health Organization and their 1983 amendments. Approval of the local ethics committee was obtained (study approval: report 8.9.0 12/2020 of the Board of the Department of Translational and Precision Medicine—Sapienza University of Rome). Informed consent was obtained from each patient.

### 2.2. Group A—Untreated Celiac Disease CD (uCD)

We enrolled 5 patients (1 male and 4 females; median age, 36.6 years; range, 20 to 63 years) on a gluten-containing diet. They self-reported gastrointestinal and extraintestinal gluten-related symptoms for at least 3 months and showed positive serological tests for CD: IgA anti-Endomysial Antibody (EMA) and IgA anti-transglutaminase (anti-tTG). Histological examination of duodenal biopsies showed villous atrophy [26].

### 2.3. Group B—Treated Celiac Disease CD (tCD)

We enrolled 5 patients (2 males and 3 females; median age, 41.2 years; range, 21 to 64 years) with previous serological and histological diagnosis of CD, on a gluten-free diet for at least 12 months. They showed serological negativization (IgA EMA and anti-tTG) and a concomitant improvement in gastrointestinal and/or extraintestinal gluten-related symptoms. Histological examination of duodenal biopsies showed mucosal recovery.

### 2.4. Group C—Wheat Allergy (WA)

We enrolled 5 patients (2 males and 3 females; median age, 38 years; range, 23 to 65 years) on a wheat-containing diet, with negative CD-specific serology, positive IgE antibodies to wheat, and self-reported gastrointestinal and extraintestinal wheat-related symptoms since at least 3 months. A flow cytometry-assisted basophil activation test (BAT) for wheat was performed to confirm the diagnosis of wheat allergy. Histological examination of duodenal biopsies showed negative results.

### 2.5. Group D—Non-Celiac Gluten Sensitivity (NCGS)

Five patients (1 male and 4 females; median age, 35 years; range, 21 to 52 years) on a gluten-containing diet with negative CD-specific serology, negative IgE antibodies to wheat, and self-reported gastrointestinal and extraintestinal gluten-related symptoms since at least 3 months were included. A dietetic assessment with an interview and a food diary has been performed by a medical doctor and an expert dietician to confirm the gluten dependence of symptoms. Specifically, the clinical evaluation has been performed by a double-blind placebo-controlled gluten challenge according to the Salerno Experts’ Criteria and a modified version of the Gastrointestinal Symptom Rating Scale (GSRS). NCGS diagnosis has been furtherly supported by gluten oral mucosa patch test positive results. Histological examination of duodenal biopsies showed negative results [18,20].

### 2.6. Group E—Nickel Allergic Contact Mucositis (Ni ACM)

Five patients (1 male and 4 females; median age, 36.2 years; range, 21 to 52 years) were included. They were on a free diet, with negative CD-specific serology, negative IgE antibodies to wheat. They reported gastrointestinal and/or extra-intestinal symptoms related to ingestion of Ni-containing foods. A specific alimentary-symptom questionnaire (GSRS) attested to their presence, as well as their severity. Ni sensitivity diagnosis has been further suggested by Ni oral mucosa patch test and LDPI positive results [24]. Histological examination of duodenal biopsies showed negative results.

### 2.7. Group F—Control Patients (CTRL)

We enrolled 5 patients (2 males and 3 females; median age, 39.4 years; range, 20 to 59 years) suffering from gastroesophageal reflux disease (GERD) and dyspepsia. They were on a free diet, with negative CD-specific serology, and negative IgE antibodies to wheat. They showed negative oral mucosa patch test results to both gluten and Ni. Histological examination of duodenal biopsies showed negative results.

### 2.8. Esophagogastroduodenoscopy with Biopsy Sampling

All patients underwent upper endoscopy to exclude or confirm CD, as a follow-up procedure in treated CD or MRGE. Four to 6 biopsy samples have been taken from the duodenum (3 or 4 from the second portion of duodenum distal to the papilla and at least 1 from the duodenal bulb) using a standard 5-mm biopsy forceps, correctly oriented on acetate cellulose filters (Bio-Optica, Milan, Italy) and fixed in 10% formaldehyde [26]. One additional biopsy from the second portion of the duodenum has been collected and fixed in glutaraldehyde 2.5% [4].

### 2.9. Light Microscopy (LM)

Duodenal specimens embedded in 10% formaldehyde have been subjected to histological analysis. After orientation, inclusion in paraffin, and cutting, morphologic analysis was performed by hematoxylin and eosin (HE) staining technique. To evaluate the small intestine mucosal architecture, the histological pattern was blindly evaluated by a trained pathologist according to Marsh–Oberhuber classification [26].

A series of observations on 20 microscopic fields running from 5x to 20x magnification were performed on LM pictures. A qualitative analysis of villous and crypt morphology, as well as IELs count, were performed.

### 2.10. Transmission Electron Microscopy (TEM)

Samples were processed for the Transmission Electron Microscopy at Microscopy Laboratory “Pietro M. Motta”, Sapienza University of Rome. After fixation in glutaraldehyde 2.5% in PBS 0.1 M PH 7.4, the samples were rinsed in phosphate buffer saline (PBS), post-fixed with 1% osmium tetroxide (OsO_4_) (Agar Scientific, Stansted, UK) in PBS, and rinsed again in PBS [27].

The samples were dehydrated in ascending series of ethanol (Carlo Erba Reagenti, Milan, Italy), immersed in propylene oxide (BDH Italia, Milan, Italy) for solvent substitution, embedded in epoxy resin (Electron Microscopy Sciences, Hatfield, PA, USA), and sectioned by Leica EMUC6 ultramicrotome. Semithin sections (1 μm thick) and ultrathin sections (60–80 nm) were cut by the ultramicrotome with a diamond knife. Semithin sections were stained with HE and observed by optical microscope (Zeiss Axioskop 40), pictures were captured using a digital camera (Zeiss AxioCamMRc). Ultrathin sections were collected on copper grids (200 Mesh, Assing Rome, Italy) and stained with lead citrate; observations were carried on by transmission electron microscope (Carl Zeiss EM10, Thornwood, NY), operating at 60 kV conditions and equipped with a digital camera (AMT CCD, Deben UK Ltd., Suffolk, UK) [28].

Digital TEM pictures were analyzed by ImageJ software; morphological features considered were: microvilli length and intermicrovillar distance, JC gap width, autophagic bodies, apoptosis, altered mitochondria, lipid drops/chylomicrons, and mast cells. As regards JC, we specifically focused on TJ, AJ, and desmosomes gap width.

The ultrastructural features of the intestine structures and junctions were visualized at magnifications ranging from 5000× to 6300×. The widths and lengths of intercellular junctions and microvilli were measured. Ultrastructural analyses were performed in 10 selected tissue areas, and only epithelial structures that were precisely longitudinally sectioned were measured. At least 10 values of each type of intercellular junction/patient and at least 15 values associated with microvilli/patient were obtained. The results were presented in nm [29].

### 2.11. Statistical Analysis

Data were expressed as number (%) ± SD as appropriate. Statistical analysis of results was performed by MedCalc software (MedCalc Software 20.009version Ltd., Acacialaan 22, 8400 Ostend Belgium), quantitative parameters values were compared using ANOVA test for multiple comparisons with Bonferroni correction. Significance was defined as *p* < 0.05.

## 3. Results

### 3.1. Light Microscopy Observations

**Figure 1:**
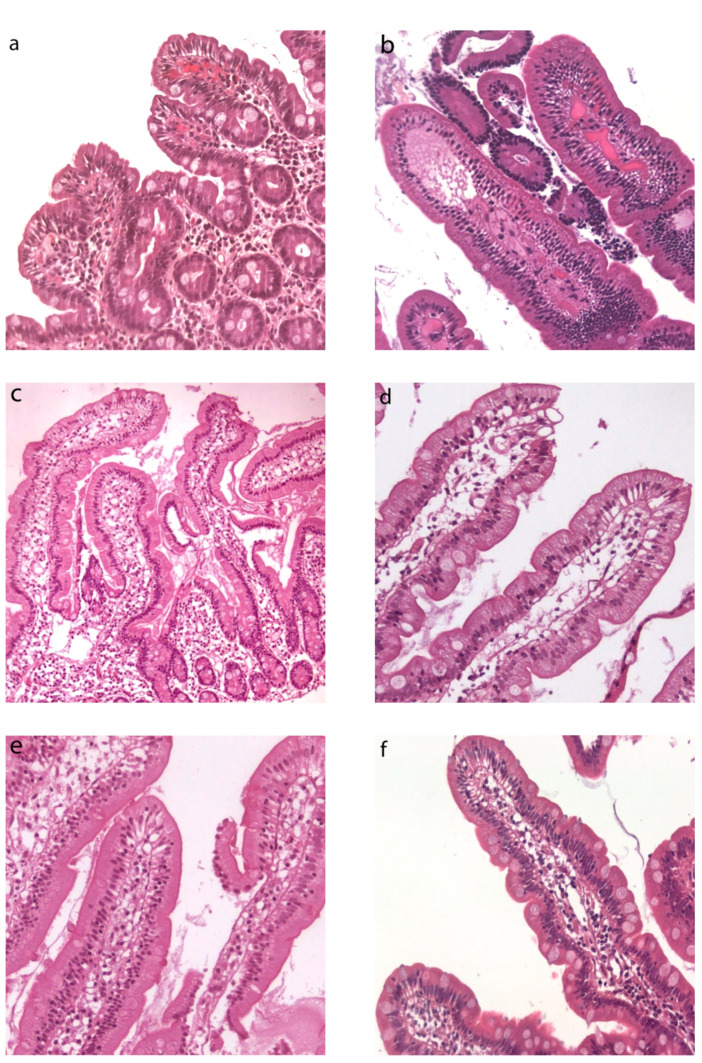
Duodenal histology from IBS-like patients. Light microscopy (LM) histological images of duodenal biopsies in IBS-like disorders (H&E, magnification 20×). Intestinal villous atrophy, crypt hyperplasia, and intraepithelial lymphocytosis in uCD patients (**a**), restitutio ad integrum in tCD (**b**), no specific alterations found in WA (**c**), NCGS (**d**), Ni ACM (**e**), as well as in controls (**f**), Legend: uCD, untreated celiac disease; tCD, treated celiac disease; WA, wheat allergy; NCGS, non-celiac gluten sensitivity; Ni ACM, Nickel allergic contact mucositis; CTRL, controls.

Duodenal histological features at LM have been summarized in Table 1. In Group A, duodenal mucosa appeared flat with hyperplasia of the crypts and intraepithelial lymphocytosis (Figure 1a). Clear duodenal mucosa healing has been observed in Group B, after a strict gluten-free diet (Figure 1b). Villous height/crypt depth ratio and intraepithelial lymphocytes were normal or showed mild specific alterations in Groups C, D, and E (Figure 1c–e). Patients from Group F did not show any significant alteration (Figure 1f).

### 3.2. Transmission Electron Microscopyobservations

Ultrastructural evaluation of duodenal biopsies from IBS-like and control patients (Groups A-F) has been summarized in Table 2.

#### 3.2.1. Length of Enterocytes Microvilli

**Figure 2:**
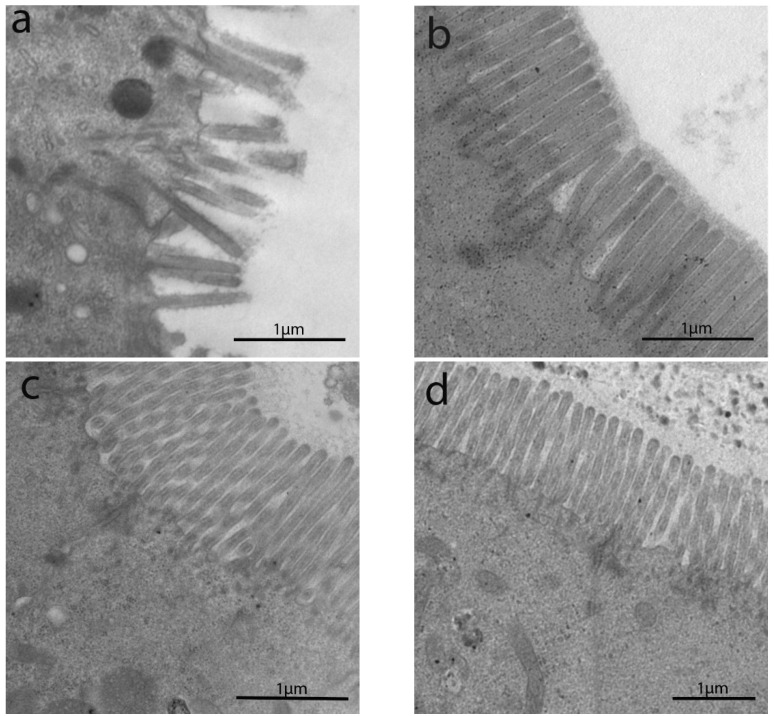

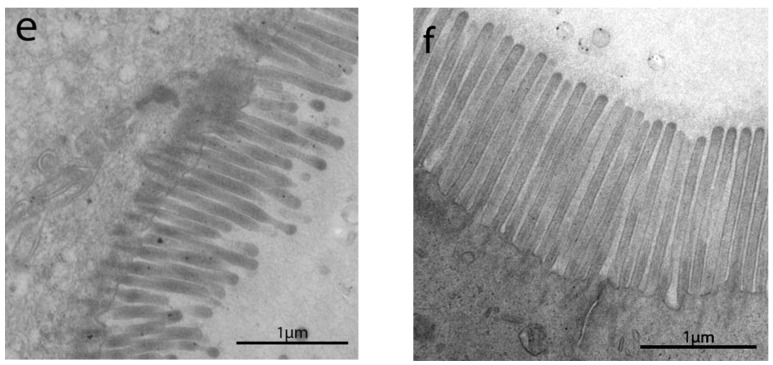
TEM images in IBS-like disorders: uCD (**a**); tCD (**b**); WA (**c**); NCGS (**d**); Ni ACM (**e**); controls (**f**). In uCD it is possible to appreciate severe atrophy of the microvilli. The restitutio ad integrum of the microvilli in tCD (**b**) is however not similar to the controls (**f**). In NCGS (**d**) and Ni ACM (**e**) microvilli appear hypotrophic and similarly distant from each other.

**Scheme 1:**
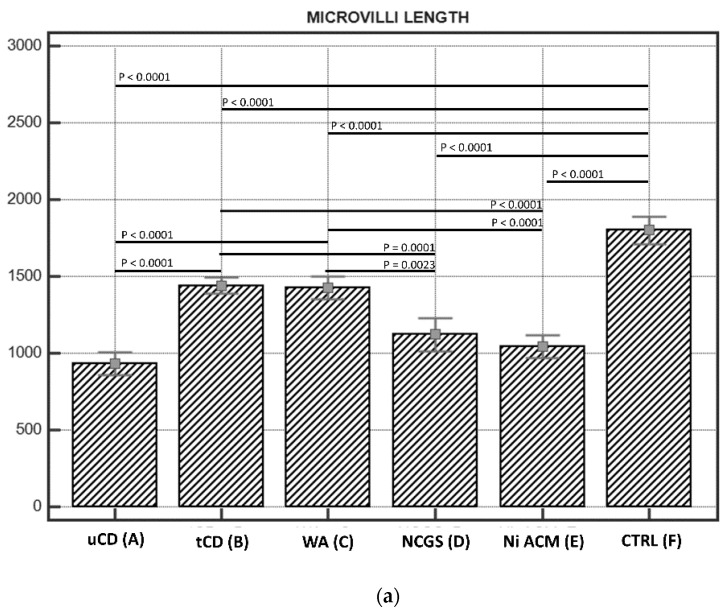

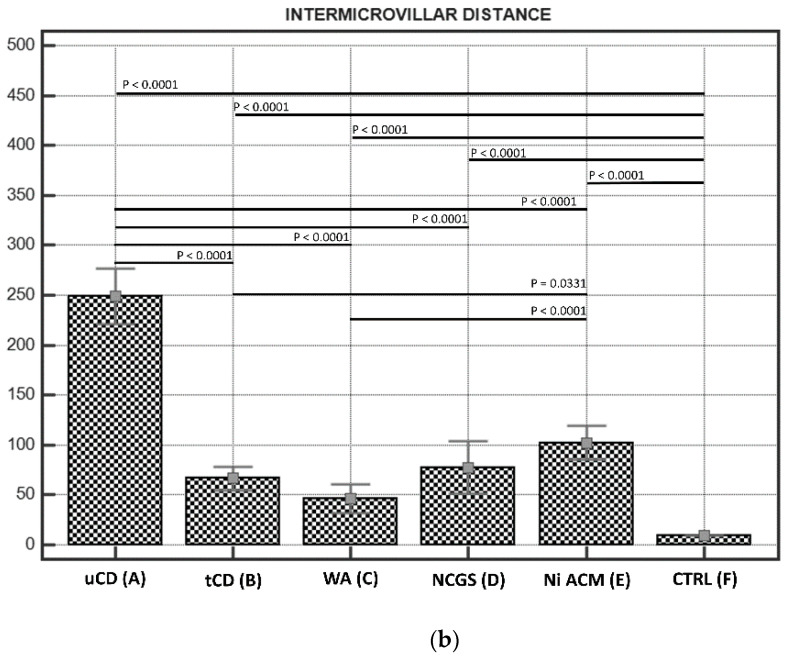
(**a**). Microvilli lengths in IBS-like disorders; the graph shows the lengths of microvilli (expressed in nm) in the IBS-like disorders studied with any significant differences: uCD; tCD; WA; NCGS; Ni ACM; controls. Persistent hypotrophic microvilli in uCD are not dissimilar to those of NCGS and Ni ACM. Microvilli in tCD appear significantly longer than in uCD, but significantly shorter than in controls. (**b**). Intermicrovillar distances in IBS-like disorders; The graph shows the intermicrovillar distances (expressed in nm) in the IBS-like disorders studied, with any significant differences: uCD; tCD; WA; NCGS; Ni ACM; controls. The most significant distances are observed in uCD; a significant recovery is observed in tCD, although the microvillar distance is smaller in the controls than in all the other groups. Similar intermediate intermicrovillar distances appear in NCGS and Ni ACM.

The length of enterocytes microvilli (Figure 2 and Scheme 1a) showed a significant decrease in all groups compared to controls. In detail, biopsies from uCD patients showed the most severe surface epithelium alteration, with partial microvilli atrophy in some portions, as well as short, irregular, and unevenly spaced microvilli in others (Figure 2a). Those irregular and sub-atrophic epithelial portions in uCD were not significantly different compared to NCGS (Figure 2d) and Ni ACM (Figure 2e) patients. A significant recovery was evidenced in tCD (Figure 2b) patients compared to uCD and showed no significant difference with WA (Figure 2c) patients. On the other hand, microvilli in tCD were significantly shorter than CTRL (Figure 2f). There was no statistically significant difference in microvilli length between NCGS (Figure 2d) and Ni ACM (Figure 2e) patients. Control patients showed no ultrastructural alteration in microvilli length (Figure 2f).

#### 3.2.2. Intermicrovillar Distance in Enterocytes

A significant increase in intermicrovillar distance of enterocyte brush border has been observed in all groups compared to controls (Figure 2 and Scheme 1b).

The most severe increase in the intermicrovillar space has been observed in uCD patients, while the narrowest space was in CTRL. Patients with CD (Figure 2b) showed a significant recovery compared to uCD (Figure 2a). On the other hand, the intermicrovillar space in tCD patients was larger than in CTRL (Figure 2f). No significant differences were found between NCGS (Figure 2d) and Ni ACM (Figure 2e), although Ni ACM revealed slightly greater intermicrovillar distances. What is more, Ni ACM revealed a significantly greater intermicrovillar distance than WA (Figure 2c) and tCD (Figure 2b). No significant difference was found among tCD (Figure 2b), WA (Figure 2c) and NCGS (Figure 2d).

#### 3.2.3. Junctional Complex in Enterocytes

**Figure 3:**
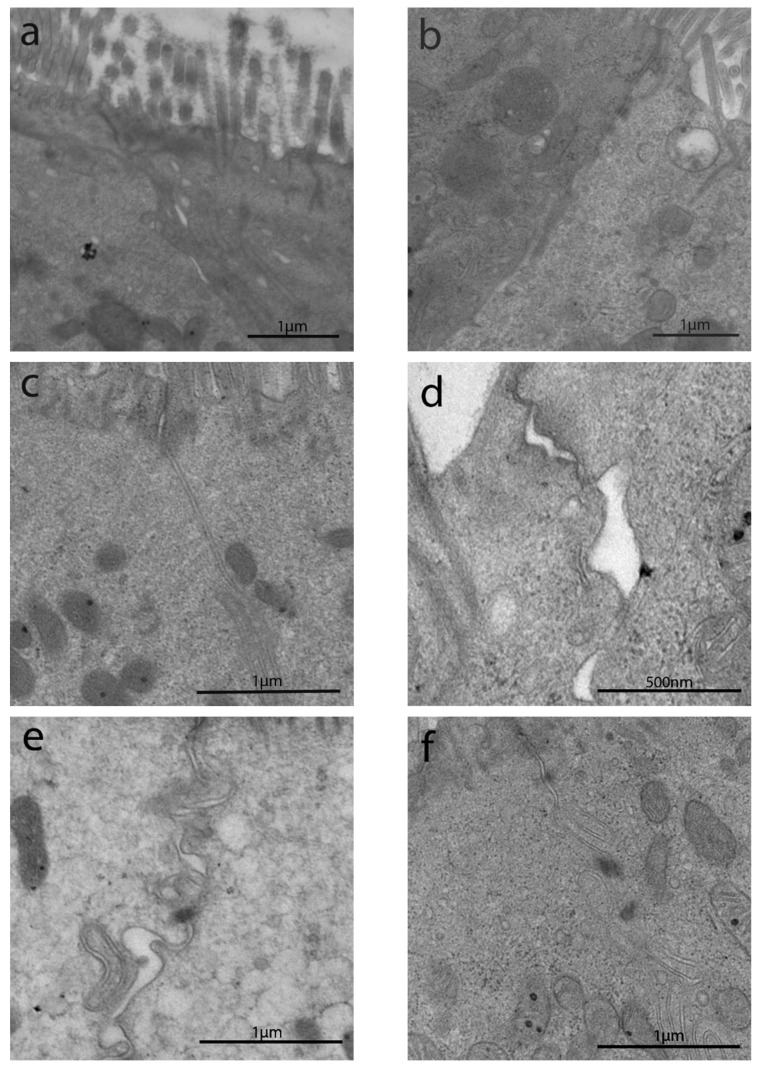
TEM images of apical junctional complexes (AJC) in IBS-like disorders: uCD (**a**); tCD (**b**); WA (**c**); NCGS (**d**); Ni ACM (**e**); controls (f). In particular, TJ gap width did not show significant differences among all groups; AJ gap was significantly more dilated in all groups compared to controls; desmosome gap was found significantly wider in uCD (**a**), NCGS (**d**) and Ni ACM (**e**) than all other groups.

**Scheme 2:**
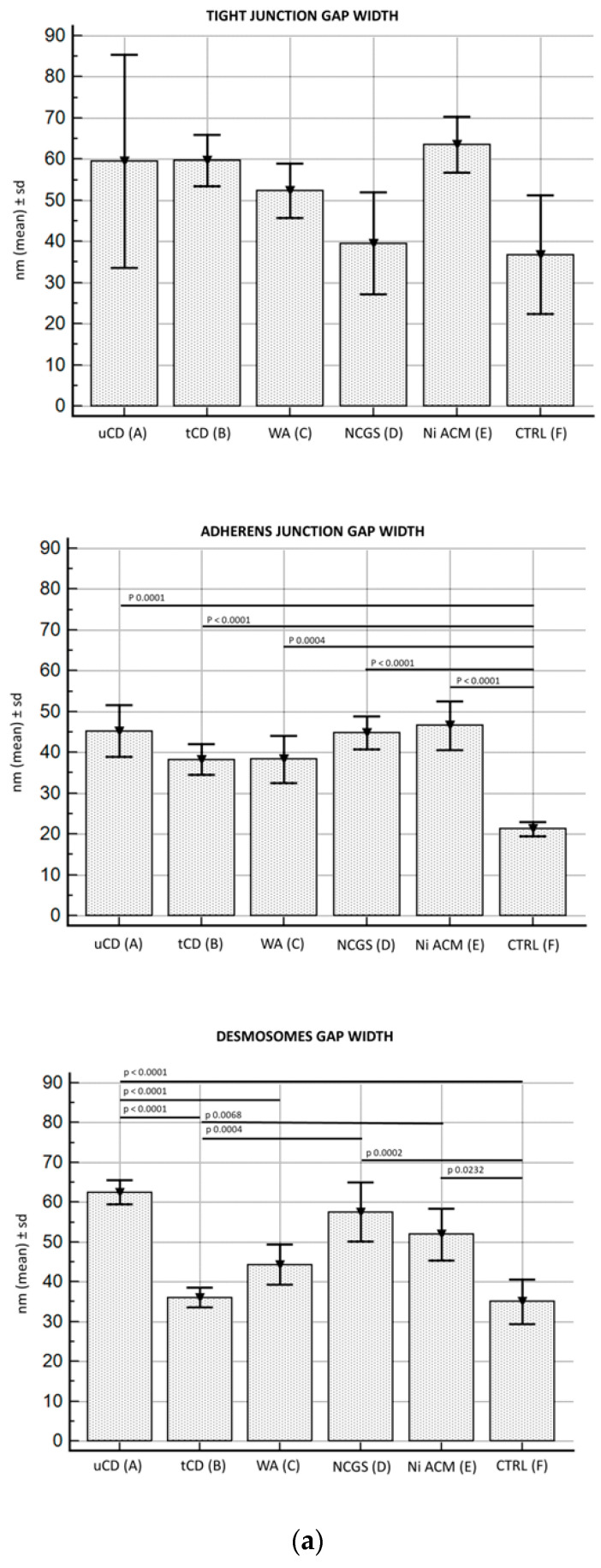

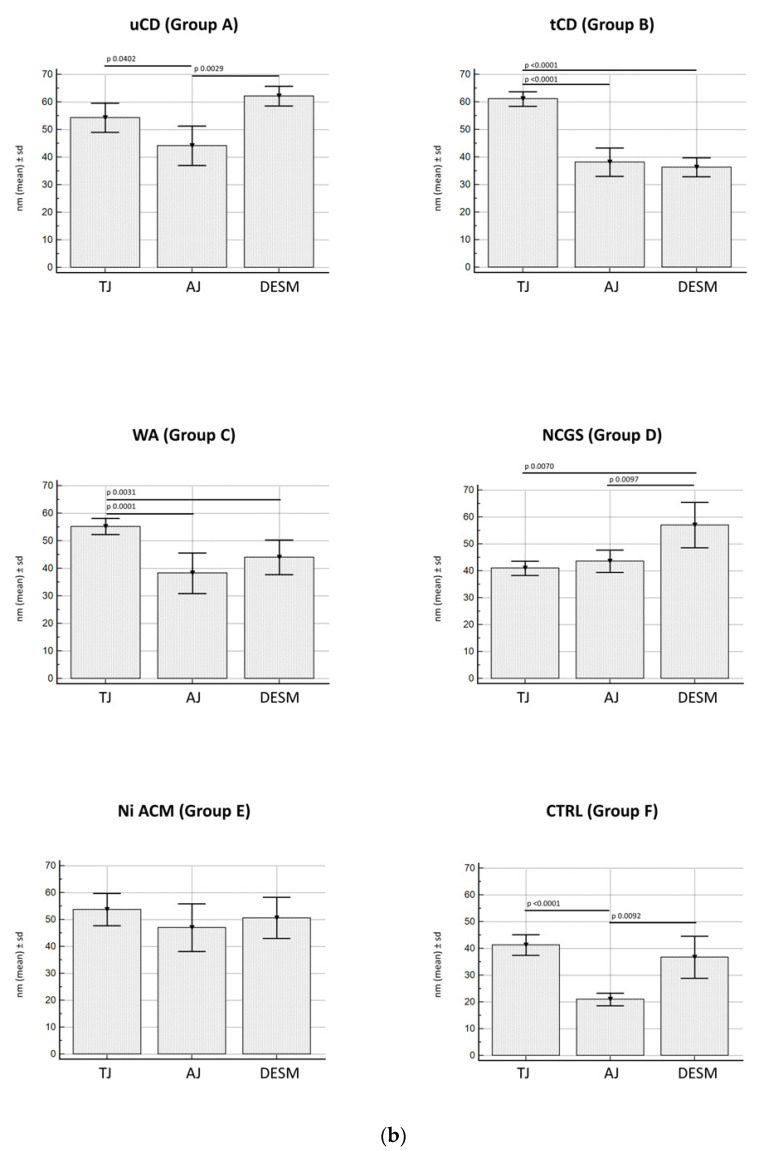
(**a**). Distances between apical junctional complexes (AJC) in the different groups under study, divided by single component (TJ, AJ, and desmosomes). Legend: uCD, untreated celiac disease; tCD, treated celiac disease; WA, wheat allergy; NCGS, non-celiac gluten sensitivity; Ni ACM, Nickel allergic contact mucositis; CTRL, controls. (**b**). Distances between the single components of the apical junctional complexes (AJC) (TJ, AJ, and desmosomes), in the different groups under study. Legend: uCD, untreated celiac disease; tCD, treated celiac disease; WA, wheat allergy; NCGS, non-celiac gluten sensitivity; Ni ACM, Nickel allergic contact mucositis; CTRL, controls.

TEM images of the JC are shown in Figure 3. The groups studied are compared according to each JC component (TJ, AJ, and desmosomes) in Scheme 2a.

Specifically, TJ gap width did not show significant differences among all groups studied, including controls, although patients with uCD showed considerable variance in data. Moreover, only a decreasing trend was found in NCGS and control patients.

AJ gap was significantly more dilated in all groups compared to controls, while no significant differences were found among the pathological groups (A–E).

The desmosome gap was found almost always significantly wider in uCD, NCGS, and Ni ACM than in tCD, WA, and control patients. No significant difference was found in the desmosome gap width among uCD, NCGS, and Ni ACM patients, although the largest distances were found in uCD and the shortest in Ni ACM. Similarly, no significant difference was observed in the desmosomes gap width among tCD, WA and controls patients.

JC components (TJ, AJ, and desmosomes) have been also compared in each group studied (Scheme 2b). In particular, uCD (Figure 3a) showed a significantly wider gap in TJ and desmosomes compared to AJ; tCD (Figure 3b) and WA (Figure 3c) patients revealed a wider TJ gap width compared to AJ and desmosomes; NCGS (Figure 3d) patients showed a significantly increased desmosomes gap width compared to TJ and AJ; Ni ACM (Figure 3e) patients showed no significant difference in JC dilatation; control (Figure 3f) patients revealed tight JCs, especially AJ compared to TJ and desmosomes.

#### 3.2.4. Intracytoplasmic Characteristics of Enterocytes

Intracytoplasmic characteristics are summarized in Table 2.

**Figure 4:**
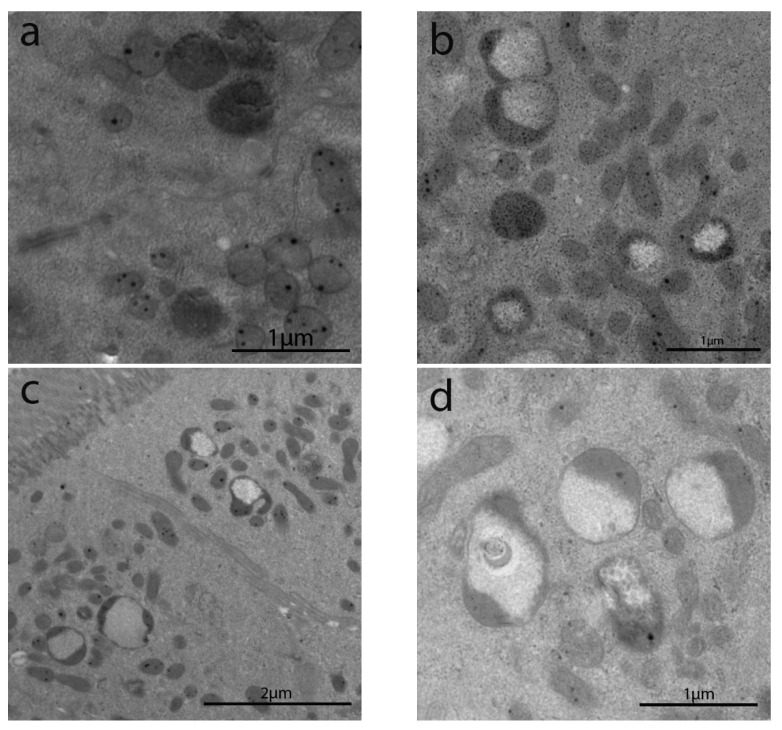

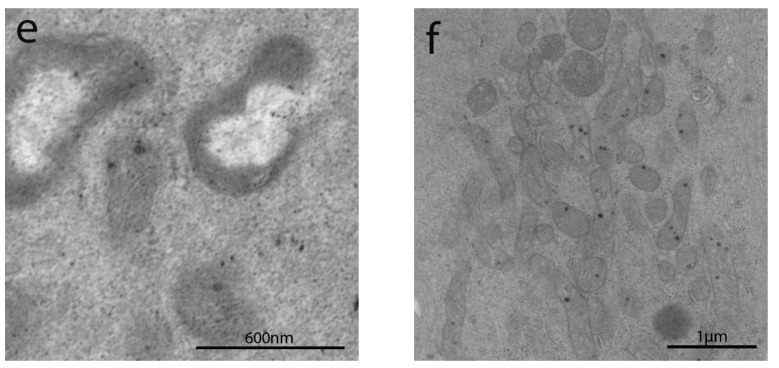
TEM images of autophagic bodies and lysosomal vacuoles in the cytoplasm of enterocytes in IBS-like disorders. Legend: uCD (**a**), tCD (**b**), WA (**c**), NCGS (**d**), Ni ACM (**e**), CTRL (**f**). All pictures (**a**–**e**) showed increased autophagic bodies, as well as lysosomal vacuoles in the apical cytoplasm, whereas no signs of autophagy have been observed in controls (**f**).

Patients from all groups showed increased autophagic bodies, as well as lysosomal vacuoles in the apical cytoplasm, whereas no signs of autophagy have been observed in control patients (Figure 4a–f).

**Figure 5:**
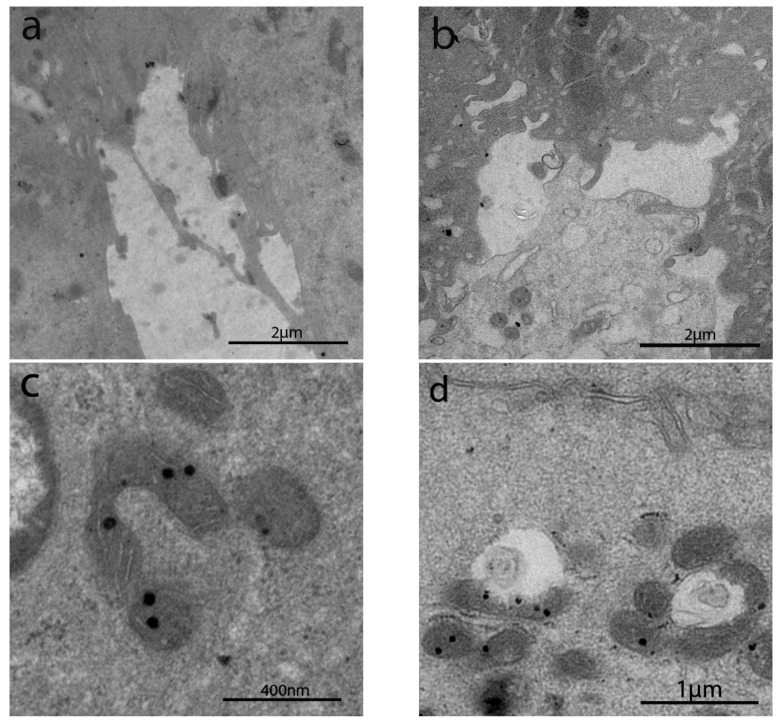

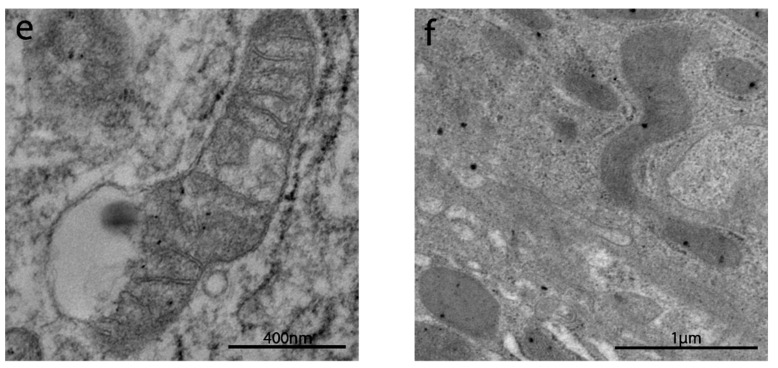
TEM images of apoptosis and mitochondria in IBS-like disorders. The sub-apical and lateral domain of enterocytes in NCGS (**a**) and Ni ACM (**b**) showed signs suggestive of apoptosis and reduction in cell turnover (**a**,**b**). Abnormal mitochondria have been observed in WA (**c**), NCGS (**d**), and Ni ACM (**e**,**f**); an irregular shape of these organelles was evident, with a swollen, flattened, or fissured matrix, in some cases associated with an increased rough endoplasmic reticulum (RER) (**e**).

**Figure 6:**
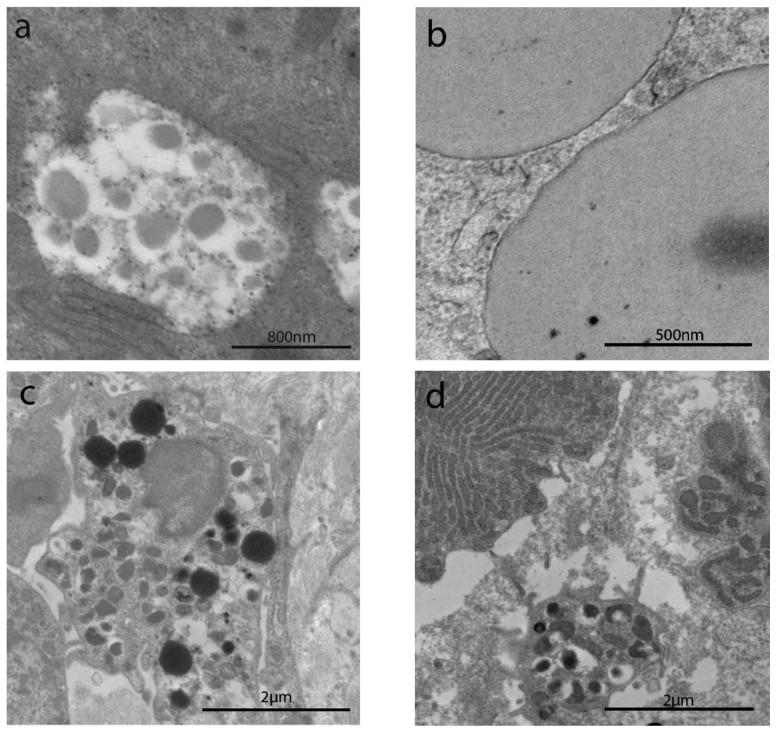
TEM images of chylomicrons, lipid droplets, and mast cells in IBS-like disorders. Chylomicrons in WA (**a**); lipid drops in NCGS and Ni ACM (**b**), mast cells in uCD, WA (**c**), and Ni ACM (**d**).

The sub-apical and lateral domain of enterocytes in NCGS and Ni ACM patients showed apoptosis and reduction in cell turnover (Figure 5a,b). Abnormal mitochondria have been observed in WA (Figure 5c), NCGS (Figure 5d) and Ni ACM (Figure 5e,f) patients (Groups C, D, E): an irregular shape of these organelles was evident, with a swollen, flattened or fissured matrix, in some cases associated with an increased rough endoplasmic reticulum (RER) (Figure 5e,f). WA patients showed many chylomicrons within the enterocytes, unlike any other group (Figure 6a). Lipid droplets have been observed in the apical enterocytes portion in NCGS (Figure 6b) and Ni ACM patients (Groups D and E).

Mast cells were detected in the subepithelial compartment in uCD (Figure 6c), WA (Figure 6d), and Ni ACM patients.

## 4. Discussion

IBS is a very common disorder that can affect more than 10% of the general population [30]. More recently, researchers have also tried to clarify the nature of IBS-like disorders, whose histopathological features are mostly unspecific and still not well understood [2]. Our study focused for the first time on IBS-like disorders from a structural and ultrastructural point of view, by performing a comparative morphometric analysis.

Preliminary morphological evaluation by light microscopy of duodenal biopsies revealed no severe alterations except for uCD patients, as expected. More specifically, all the other groups, including tCD and controls, showed no or mild/unspecific alterations compatible with Marsh–Oberhuber type 0–1. On the other hand, our data confirmed a raised IELs count >25 per 100 enterocytes in about half of the NCGS patients as described in the literature [31]. Further investigations with immunohistochemistry could provide data and explanations on this topic.

Our observation by TEM has been initially dedicated to the presence of microvilli on the luminal surface of enterocytes and their features, which have been observed and described for the first time by Granger and Baker using electron microscopy [32]. Alterations in the structure of intestinal microvilli include an irregular arrangement, shortening, widening and fusion of microvilli in uCD, thus reducing the absorptive intestinal surface [33,34,35]. Other authors also described short, irregular, and loosely packed microvilli in uCD [16]. On the other hand, the fusion of microvilli seems to be a common response also to other acute and chronic stressful conditions [36].

Ultrastructural alterations regarding microvilli length and intermicrovillar distance have been observed in almost all pathological groups in our study, although with different degrees of impairment.

Our data confirmed both total atrophy and sub-atrophy of intestinal microvilli in uCD patients, according to an alternating pattern [29]. Similar sub-atrophic microvilli have also been found in NCGS and Ni ACM patients, although there were no areas of total atrophy in these last two conditions: these microvillous alterations in NCGS and Ni ACM could represent an early manifestation of a non-IgE mediated intolerance/allergy which can cause gastrointestinal symptoms but not necessarily a malabsorption syndrome. Moreover, a proper GFD appeared to induce a significant elongation of microvilli in tCD, although they remained shorter than in controls: this could justify the persistent symptoms that CD patients often complain of, despite a proper GFD [37].

Since previous ultrastructural analysis already showed altered intercellular junctions and intercellular spaces in uCD [29], we wanted to confirm similar features in the most frequent IBS-like disorders.

Unlike Sowińska et al. who described more minor TJ gap in uCDs than in controls, TJ gap width in our cases did not show significant differences between uCD and controls, although values in uCD tended to be higher. The great variance of our data could explain this disagreement. On the other hand, we confirmed significantly wider AJ in uCD compared to controls. We also found significantly wider desmosomes gap width in uCD compared to controls, whereas Sowińska et al. described a simple incorrect/asymmetrical structure of desmosomes in uCD and wider desmosomes gap only in potential CD [29].

We also observed that in all IBS-like disorders, including tCD, the AJ gap width was similar to uCD and significantly increased compared to controls.

On the other hand, the largest gap between the desmosomes has been found not only in uCD (as expected) but also in NCGS and Ni ACM: there was no significant difference among these three groups, although higher values were found in uCD and lower values in Ni ACM.

These observations could suggest two pathological JC arrangement models in IBS-like disorders: (1) the “hourglass” model for uCD, WA, and Ni ACM, where the greatest dilations involve both TJ and desmosomes; (2) the “semi-open zipper” model for NCGS, where a single and large breach only concerns the desmosomes.

These intestinal barrier ultrastructural alterations involving CJ, as well as microvilli, are here for the first time described in IBS-like disorders and can be responsible for increased intestinal permeability, as well as IBS-like symptoms.

Immune-histochemical staining and Western blot analysis of CJ proteins may be performed in future studies to confirm these significant ultrastructural alterations: these changes may probably be a consequence of overexpression of pore-forming proteins and under-expression of pore-sealing proteins triggered by wheat, gluten or Ni [4].

Macroautophagy or autophagy is a process to maintain cellular homeostasis: its regulatory mechanisms are still not well-known, but they seem to have a role in many disorders. Specifically, many works showed a possible role of autophagy in eliminating intracellular bacteria and viruses [38,39]. Autophagy is also probably involved in many other intestinal mucosa responses to stress, even in inflammatory bowel diseases. Defects in autophagy may result in increased auto-reactive T cells within the gut. Moreover, a link between autophagy and regulation of cell-cell junctions has been observed, thus a defect in autophagy may affect intestinal permeability [40]. Our results confirm the high number of autophagic bodies selectively present in the apical position of intestinal cells, as well as numerous autophagosomes in all experimental groups except in control patients. This evidence, in agreement with Barone et al., may support the hypothesis that gluten, as well as Ni, may influence cell inflammatory signaling and trigger stress-dependent molecular pathways such as autophagy [41].

Moreover, autophagy has probably a role together with apoptosis: autophagy acts as a cytoprotective mechanism, but if the cellular damage is too severe and/or apoptosis is compromised, excessive autophagy may be used to kill the cell [39]. Infectious and other stress-related conditions may alter the normal modulatory mechanisms of enterocyte apoptosis and the homeostatic turnover of the epithelial layer, resulting in increased epithelial permeability and chronic gastrointestinal disorders, such as IBS-like disorders and IBD [42]. Significantly higher level of apoptosis has been described in uCD patients so far, despite its precise role is still unclear [43,44]. We did not confirm the presence of apoptosis in our case series of uCD patients, but patients suffering from Ni ACM and NCGS showed a marked cell loss and apoptosis: this may negatively influence intestinal homeostasis may because of damage to epithelial permeability.

Mitochondria not only produce cellular energy but also regulate other vital cellular processes, such as the induction of programmed cell death, transduction of stress, and maintenance of the epithelial barrier [45]. In WA, NCGS and Ni ACM groups mitochondrial ultrastructure showed many alterations, with swollen organelles, often accompanied by loss of cristae ridges and electron-dense inclusions within the mitochondrial matrix. These kinds of abnormalities have also been described in IBD [45] and l chronic alcoholic patients [46].

In the biopsies of patients with Ni and gluten sensitivity, large cytosolic lipid drops have been observed. Unfortunately, little is known about the mechanisms that control the distribution of lipids in the enterocytes and the consequences of their alteration on the pathophysiology. It has been supposed that alteration of proteins associated with these lipid drops can affect the synthesis of chylomicrons, reducing lipid absorption from the lumen and altering the gut microbiota: this could lead to the onset of gastrointestinal disorders [47].On the other hand, lipid drops are also considered to have an active role in inflammation, not only in the uptake of lipopolysaccharide (LPS) but also in the assembly and replication of viruses and the production of inflammatory mediators. Intracellular lipid pools can also be regulated by the already mentioned autophagy pathway induced by stress, nutrient starvation, or infection, which is crucial for cellular homeostasis [48]. All these conditions related to lipid drops may be present in the IBS-like disorders studied and explain the clinical alterations in these patients.

Mast cells are involved in many conditions such as chronic allergic/inflammatory disorders [49]. They are also considered important modulators of gut homeostasis in humans since their presence and activity seem related to epithelial integrity, endothelial functions, immune regulation, microbial defense, neurological activity, and tissue transformation. So far, the specific mechanisms of these processes remain unclear [50]. Accumulation of mast cells has been described in duodenal specimens of uCD patients in the literature and their number seems to also increase with the extent of histological damage [51]. In 1962, Hiatt and Katz were the first to report an increase in the number of mast cells in muscolaris mucosae in patients with “spastic colitis” [52], and more recently, mast cells appeared also to be increased in the small intestine of IBS-like patients compared to controls [53,54]. Other authors even described possible neuro-immune interactions in the duodenal submucosa of NCGS patients involving mast cells and their close vicinity to nerves in order to explain GI symptoms [55]. Our data seem to confirm the presence of mast cells in uCD as a chronic inflammatory autoimmune disorder and their regression following a proper GFD. An IgE–mast-cell axis is well known in allergic-related chronic inflammation: the persistent exposure to allergens can result in an increase in mast cell number [56]. This should justify the presence of mast cells among our WA patients. What is more, we also found mast cells in Ni ACM [25]: this could confirm theories about the possible coexistence of type I and type IV hypersensitivity to Ni [57,58,59]. No mast cells in samples from NCGS patients have been observed, but we cannot exclude that NCGS may be a “non-IgE-mediated wheat allergy” involving mast cells, eosinophils, and other immune cells [31].

## 5. Conclusions

TEM made it possible to identify subcellular anomalies not otherwise observable by optical microscopy: significant alterations seem to involve not only the distribution and appearance of intestinal microvilli but also the intermicrovillar distance. As expected, major alterations have been observed in the subjects affected by uCD. Microvilli of tCD patients showed marked recovery after adequate GFD, although not comparable to controls. Intermediate microvillar alterations were instead observed in NCGS and Ni ACM, while characteristics of WA subjects appeared more similar totCD.

Intracellular alterations seemed more unspecific, but probably attributable to low-grade inflammation or adverse reaction triggered by food allergens (gluten/wheat), heavy metals (Ni), or other unknown. These changes may be defense mechanisms activated by enterocytes in response to damage to the intestinal barrier.

It was a pilot study conducted in a single center and many observations or measurements have never been performed before in such categories of patients: these are significant limits. Thus, further investigations with larger populations will be needed to confirm our preliminary observational data. Furthermore, immunohistochemical, molecular, and intestinal permeability investigations may be extremely useful to eventually validate what has been observed, to clarify the link between ultrastructural alterations and signs/symptoms in IBS-like patients and support diagnostic and therapeutic facilitation.

## Figures and Tables

**Table 1:** Morphological evaluation by light microscopy of duodenal biopsies in IBS-like patients.

Groups	Villous:Crypt Ratio	Crypts	IELs/100 ECs	Marsh-Oberhuber Classification
(A) **uCD**				
pt n.1	1:3	hyperplasia	≥25	3 C
pt n.2	1:2	hyperplasia	≥25	3 B
pt n.3	1:3	hyperplasia	≥25	3 C
pt n.4	1:2	hyperplasia	≥25	3 A
pt n.5	1:2	hyperplasia	≥25	3 B
(B) **tCD**				
pt n.1	3:1	normal	<25	0
pt n.2	3:1	normal	≥25	1
pt n.3	3:1	normal	<25	0
pt n.4	2:1	hyperplasia	≥25	2
pt n.5	2:1	normal	≥25	1
(C) **WA**				
pt n.1	3:1 (with dismorphyc villi)	normal	≥25	1
pt n.2	3:1	normal	<25	0
pt n.3	3:1 (with dismorphyc villi)	normal	≥25	1
pt n.4	3:1	normal	<25	0
pt n.5	3:1	normal	<25	0
(D) **NCGS**				
pt n.1	3:1	normal	<25	0
pt n.2	3:1	normal	<25	0
pt n.3	3:1 (with dismorphyc villi)	normal	≥25	1
pt n.4	3:1	normal	<25	0
pt n.5	3:1 (with dismorphyc villi)	normal	≥25	1
(E) **Ni ACM**				
pt n.1	3:1 (with dismorphyc villi)	normal	≥25	1
pt n.2	3:1	normal	<25	0
pt n.3	3:1	normal	<25	0
pt n.4	3:1	normal	<25	0
pt n.5	3:1 (with dismorphyc villi)	normal	≥25	1
(F) **CTRL**				
pt n.1	3:1	normal	<25	0
pt n.2	3:1	normal	<25	0
pt n.3	3:1	normal	<25	0
pt n.4	3:1	normal	≥25	1
pt n.5	3:1	normal	<25	0

**Table 2:** Ultrastructural evaluation by electron microscopy of duodenal biopsies in IBS-like patients A qualitative score (++ = marked presence; + = presence; - = absence) was provided for widened junctional complexes, autophagy, apoptosis, altered mitochondria, lipid drops, chylomicrons, and mast cells. Legend: CTRL, control patients; JC, junctional complexes; NCGS, non-celiac gluten sensitivity; Ni ACM, Nickel allergic contact mucositis; tCD, treated celiac disease; uCD, untreated celiac disease; WA, wheat allergy. * microvilli length and inter-microvillar distance in uCD patients have been measured in those portions of epithelium surface not involved by total microvillar atrophy.

Transmission Electron Microscopy													
Groups	Gender	Age	Symptoms	Microvilli			JC	Auto Phagy	Apo Ptosis	Altered Mitochondria	Lipid Drops	Chylomicrons	Mast Cells
				Length	Distance	Regularity							
**(A) uCD**				931.00 ± 30 *	249 ± 10 *								
**pt n.1**	F	20	diarrhea, anemia			atrophic/irregular and loosely packed	see graph	++	-	-	-	-	++
**pt n.2**	F	63	constipation, epigastralgia, heartburn			atrophic/irregular and loosely packed		++	-	-	-	-	+
**pt n.3**	M	34	Abdominal swelling, diarrhea			atrophic/irregular and loosely packed		++	-	+	-	-	-
**pt n.4**	F	25	abdominal pain, swelling, heartburn, asthenia			atrophic/irregular and loosely packed		+	-	-	-	-	++
**pt n.5**	F	41	abdominal pain, swelling, nausea, vomiting, headache			atrophic/irregular and loosely packed		++	-	-	-	-	+
**(B) tCD**				1439.10 ± 69	66.55 ± 7								
**pt n.1**	F	51	asymptomatic			regular butlooselypacked	See graph	++	-	+	-	-	-
**pt n.2**	M	39	acid regurgitation, headache			regular and tightly-packed		++	-	-	-	-	-
**pt n.3**	F	21	asymptomatic			regular and tightly-packed		+	-	-	-	-	-
**pt n.4**	F	31	asymptomatic			regular and tightly-packed		++	-	-	-	-	-
**pt n.5**	M	64	abdominal swelling, flatulence			regular and tightly-packed		+	-	-	-	-	-
**(C) WA**				1427.09 ± 71	46.23 ± 8								
**pt n.1**	F	23	diarrhea, abdominal pain, swelling, anemia, headache			regular and packed	see graph	++	-	+	-	+	-
**pt n.2**	F	28	abdominal pain, diarrhea			regular and packed		++	-	+	-	+	+
**pt n.3**	F	41	diarrhea, abdominal swelling, atopic dermatitis			regular and tightly-packed		++	-	-	-	+	+
**pt n.4**	M	65	diarrhea, abdominal bloating, asthenia, headache			regular and packed		++	-	+	-	+	+
**pt n.5**	M	33	Abdominal swelling, asthenia			regular and tightly-packed		++	-	+	-	-	+
**(D) NCGS**				1120.69 ± 60	77.59 ± 12								
**pt n.1**	F	52	abdominal pain, swelling, diarrhea, asthenia			regular and packed	see graph	+	+	+	+	-	-
**pt n.2**	M	35	abdominal pain, swelling			regular and packed		+	+	+	-	-	-
**pt n.3**	F	38	heartburn, nausea, headache, foggy mind			regular and loosely-packed		++	+	+	+	-	-
**pt n.4**	F	28	abdominal pain, swelling, asthenia, alopecia			regular and packed		+	-	+	+	-	-
**pt n.5**	F	22	abdominal pain, swelling, diarrhea			regular and tightly-packed		++	+	-	+	-	-
**(E) Ni ACM**				1042.29 ± 42	102.30 ± 10								
**pt n.1**	M	33	abdominal pain, diarrhea, heartburn			irregular and packed	see graph	++	+	+	+	-	+
**pt n.2**	F	21	abdominal pain, swelling, constipation			regular and packed		+	-	+	-	-	-
**pt n.3**	F	30	abdominal pain, swelling			irregular and packed		++	+	+	+	-	+
**pt n.4**	F	45	abdominal pain, swelling, constipation			irregular and packed		++	+	+	+	-	+
**pt n.5**	F	52	abdominal pain, swelling, diarrhea, asthenia, anemia			irregular and packed		++	+	+	+	-	+
**(F) CTRL**				1799.75 ± 49	9.6 ± 1								
**pt n.1**	F	20	GERD and dyspepsia			regular and tightly-packed	see graph	-	-	-	-	-	-
**pt n.2**	F	30	GERD and dyspepsia			regular and tightly-packed		-	-	-	-	-	-
**pt n.3**	M	45	GERD and dyspepsia			regular and tightly-packed		-	-	-	-	-	+
**pt n.4**	F	59	GERD and dyspepsia			regular and tightly-packed		-	-	-	-	-	-
**pt n.5**	M	43	GERD and dyspepsia			regular and packed		+	-	-	-	-	-

## Data Availability

Micrographs and tables are stored in our University, Sapienza University of Rome and are available upon request.
